# Fibrodysplasia ossificans progressiva with two emergency laparotomies: A case report

**DOI:** 10.1016/j.ijscr.2020.07.033

**Published:** 2020-07-16

**Authors:** Nobuyuki Okamoto, Tatsuya Tazaki, Ryuta Shintakuya, Toshinori Hirano, Masaru Sasaki, Shinya Takahashi, Astushi Nakamitsu

**Affiliations:** a Department of Surgery, JA Hiroshima General Hospital, Hiroshima, Japan; b Department of Surgery, Graduate School of Biomedical and Health Sciences, Hiroshima University, Hiroshima, Japan

**Keywords:** Fibrodysplasia ossificans progressiva, Heterotopic ossification, General surgery, Case report

## Abstract

**Introduction:**

Fibrodysplasia ossificans progressiva (FOP) is a disorder causing progressive heterotopic ossification of muscles, tendons, and ligaments. Invasive procedures such as surgery should be avoided, because physical stimulation causes heterotopic ossification.

**Presentation of case:**

A 40-year-old Japanese man with FOP was transported to our hospital with sudden abdominal pain. Emergency surgery was performed because a computed tomography scan showed the presence of intraabdominal free air. We diagnosed peritonitis due to perforation of Meckel's diverticulum and performed a small intestinal resection. The day after surgery, airway obstruction was recognized, and tracheostomy was required. Six months after surgery, a strangulated small bowel obstruction developed, and a second laparotomy was performed. As the patient continued to have difficulty swallowing, we constructed a gastrostomy at the time of the second surgery. He was discharged with no complications.

**Discussion:**

Ossification of the abdominal incision wound due to surgical invasion was suspected, but it did not occur in the short term.

**Conclusion:**

Two laparotomies could be performed safely in a patient with FOP.

## Highlights

• We underwent two emergency laparotomies for FOP patient.
• Ossification of the incision did not occur within 6 months.
• Two laparotomies could be performed safely in a patient with FOP.

## 1 Introduction

Fibrodysplasia ossificans progressiva (FOP) is a rare genetic disorder causing progressive heterotopic ossification (HO) of muscles, tendons, and ligaments [1,2]. Invasive procedures such as injections, biopsies, and surgery should be avoided, because physical stimulation causes HO [3]. We report a patient with FOP who underwent two laparotomies in about 6 months. This case is reported according to the Statement Updating Consensus Surgical Case Report (SCARE) guidelines [4].

## 2 Presentation of case

A 40-year-old Japanese man had been diagnosed with FOP at the age of 5 years. He had no family members with FOP. He had widespread joint and muscle ossification and immobility. However, since his fingers could move, he could use aids such as walking sticks to perform his daily life activities.

**Fig. 1:**
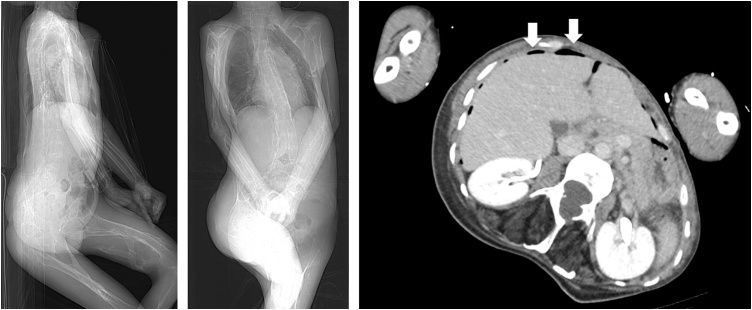
Abdominal CT image prior to surgery showed the presence of intraabdominal free air. The patient’s limbs were hardened and unable to extend.

**Fig. 2:**
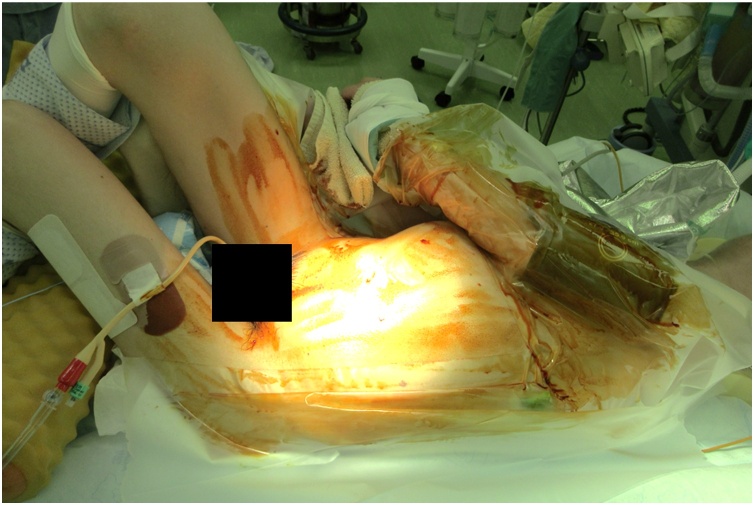
Surgical position.

He was transported to our hospital with sudden abdominal pain. Assessment by computed tomography (CT) scan showed intraabdominal free air (Fig. 1). We diagnosed his condition as perforated peritonitis and performed emergency surgery. We had difficulty positioning him because his limbs were hardened and unable to extend. The abdomen was exposed as much as possible (Fig. 2), and we were able to perform the surgery.

We were able to initiate the laparotomy as usual with an electric scalpel. However, his abdominal wall remained stiff, even after muscle relaxant administration, making it difficult to explore the abdominal cavity. We found a perforation of Meckel's diverticulum and performed a partial resection of the small intestine. When closing the wound, we found it difficult to contain the fascia. The fascia was closed with a monofilament-absorbing thread and a single ligature suture, and then the skin was closed. We did not perform extubation in the operating room, because the tension of the closed wound was so high that we feared that the suture would tear due to coughing during removal of the endotracheal tube.

The following day, the endotracheal tube was removed. However, the patient was soon re-intubated due to difficulty in expectorating sputum. Upper airway obstruction was confirmed at the time of reintubation, and a tracheostomy was performed. Because of his compromised swallowing ability, nasal tube feeding was performed. The patient was discharged after rehabilitation.

**Fig. 3:**
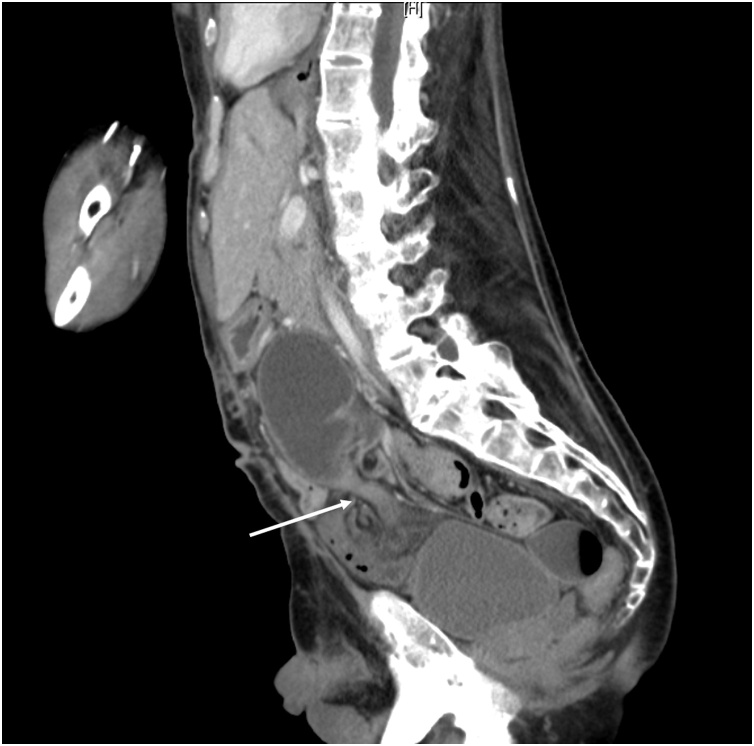
Sagittal section of abdominal CT image prior to the second surgery showed small bowel stenosis and no ossification in the previous surgical scar.

Six months after the initial surgery, the patient had sudden abdominal pain and vomiting and was transported to our hospital. We diagnosed the condition as strangulated small bowel obstruction and performed an emergency laparotomy. Preoperative CT showed no ossification in the previous surgical scar (Fig. 3). The surgical position and skin incision were the same as before. No ossification of the wound was observed, and we were able to open it without a problem. A cord formed by an adhesion between the greater omentum and the retroperitoneum caused intestinal obstruction, and we removed the cord and relieved the intestinal obstruction. Because the patient continued to have difficulty swallowing, we constructed a gastrostomy. We had difficulty closing the abdomen, but we were able to close it in the same way as before. The patient was discharged on the 19th postsurgical day with no complications and has remained well up to the time of this writing (about 3 months after the operation).

## 3 Discussion

The worldwide prevalence of FOP is estimated at approximately one in two million. The median estimated lifespan of individuals with FOP is 56 years. During the first decade of life, most children with FOP develop episodic, painful inflammatory soft tissue swellings (flare-ups). While some flare-ups regress spontaneously, most transform soft connective tissues, including aponeuroses, fascia, ligaments, tendons, and skeletal muscles, into mature heterotopic bone [5]. Most patients are confined to a wheelchair by the end of the second decade of life and commonly die of complications of thoracic insufficiency syndrome [6].

Very few reports [7,8] have described laparotomy for FOP. Moreover, there are no reports of a patient undergoing laparotomy twice.

Furuya reported that in FOP, only skeletal muscle is ossified, with smooth muscle and cardiac muscle are not. Consequently, swallowing and cardiac function are not often affected [9]. However, our patient had worsening swallowing function after the first surgery, making it difficult for him to ingest solid foods. While we were considering indications for percutaneous endoscopic gastrostomy, the patient required a second surgery, so we performed gastrostomy during the second surgery.

Pignolo et al. found that HO is associated with FOP [3]. HO has been reported in several cases as a condition in which bone tissue forms at the location of nonossified soft tissue outside the skeleton [10,11]. Jacobs et al. retrospectively reviewed 11 patients with HO and reported that initial time to ossification after surgery ranged from 11 days to 36 months (mean 6.8 months) [12]. In our patient, it was anticipated that after laparotomy, ossification of the incision would occur, making another surgery difficult. However, ossification did not occur within 6 months, so the second laparotomy could be performed without any problems. It should be noted that in the perioperative period, we tried to minimize invasive procedures, such as blood sampling, to prevent ossification. No ossification of the incision site was observed.

In the future, it is anticipated that the life expectancy of patients with FOP will be prolonged due to the progress of medical treatment, and the number of cases requiring laparotomy will increase. Therefore, it is necessary to obtain information regarding the postsurgical progress of patients with FOP.

## 4 Conclusion

Ossification of the abdominal incision due to surgical invasion was suspected, but it did not occur in the short term.

## Declaration of Competing Interest

All authors have no conflicts of interest.

## Funding

This research did not receive any specific grant from any funding agency.

## Ethical approval

The approval of our institutional ethics committee is unnecessary for a clinical case report.

## Consent

Written informed consent was obtained from the patient for publication of this case report and accompanying images. A copy of the written consent is available for review by the Editor-in-Chief of this journal on request.

## Author contribution

Study conception and design: Okamoto.

Surgical team: Tazaki, Shintakuya, Hirano.

Critical revision of manuscript: Sasaki, Takahashi, Nakamitsu.

All authors have read and approved the final manuscript.

## Registration of research studies

This case report was not registered.

## Guarantor

Nobuyuki Okamoto.

## Provenance and peer review

Not commissioned, externally peer-reviewed.
